# Supplementary material for: SVIP is a molecular determinant of lysosomal dynamic stability, neurodegeneration and lifespan
Source: Nat Commun. 2021 Jan 21;12:513. doi: 10.1038/s41467-020-20796-8 (PMC7820495; doi:10.1038/s41467-020-20796-8)
Supplement: Supplementary file 1 — Supplementary Information [file 41467_2020_20796_MOESM1_ESM.pdf]

SUPPLEMENTARY DATA

SVIP is a Molecular Determinant of Lysosomal Dynamic Stability, Neurodegeneration and Lifespan

Alyssa E. Johnson[1,2], Brian O. Orr[1], Richard D. Fetter[1], Armen J Moughamian[1,3], Logan A. Primeaux[2], Ethan G. Geier[4], Jennifer S. Yokoyama[4], Bruce L. Miller[4] and Graeme W. Davis[1*]

1. Department of Biochemistry and Biophysics
Kavli Institute for Fundamental Neuroscience
University of California, San Francisco
San Francisco, CA 94158
Graeme.davis@ucsf.edu
*to whom correspondence should be addressed

2. Current Address:
Department of Biological Sciences
Louisiana State University
Baton Rouge, LA, 70803

3. Department of Neurology
University of California, San Francisco
San Francisco, CA 94158

4. Department of Neurology, Memory and Aging Center
University of California, San Francisco
San Francisco, CA 94158

Number of Supplemental Figures: 8

Supplemental methods:

Insoluble Protein fractionation

Whole adult flies were flash frozen in an ethanol/dry ice bath and lysed directly into NP40 buffer: 6mM Na_2HPO_4, 4mM NaH_2PO_4, 1% NP40, 150mM NaCl, 2 mM EDTA, 50mM NaF, 0.1mM Na_3VO_4, 4ug/ml leupeptin and a complete protease inhibitor tablet (Roche), pH 7.4. Lysis was performed using a bug buster bead beater at room temperature. Lysates were cleared by spinning at low speed and extracted into new 1.5 ml tubes. Insoluble proteins were fractionated by centrifuging protein lysates at 20,000 g for 1.5 hrs at 4C. The insoluble protein pellet was dissolved in detergent insoluble buffer (8mM Urea, 2% SDS, 50mM DTT and 50mM Tris, pH 8) and further denatured by boiling for 10 min. Proteins were resolved by SDS-PAGE on a 4–12% Bis-Tris gel (Thermo Fisher), transferred to a nitrocellulose membrane, immunoblotted with primary and fluorescently labeled secondary antibodies and detected using a Chemidoc imager (Biorad). The following primary antibodies were used: anti-Ref(2)P (kind gift from Dr. Gabor Juhasz [1]) at 1:1000 and anti-Tubulin E7-c (Developmental Studies Hybridoma Bank) at 1:10,000. HRP conjugated secondary antibodies (Thermo Fisher) were used at 1:2000.

Western blotting

5 adult flies were grinded directly in 5X sample buffer (1M Tris-HCl, pH 6.8, 1mM DTT, 20 %SDS, 60% Glycerol, Bromophenol Blue). Proteins were resolved on a 4-12% Bis-Tris gel with either MOPS or MES running buffer (Invitrogen, Thermo Fisher) and transferred to a nitrocellulose membrane for immunoblotting. Primary antibodies used include anti-SVIP (generated in this study) used at 1:2000, anti-Tubulin E7-c (Developmental Studies Hybridoma Bank) used at 1:10,000, GPR78 monoclonal antibody (Invitrogen; MA5-27687) used at 1:1000

and actin monoclonal antibody (ACTN05) (C4), Invitrogen) used at 1:1000. The SVIP antibody was raised against recombinant GST-SVIP in guinea pig (Cocalico) and specificity was tested using the *SVIP*^{KO} mutant. Fluorescent or HRP-conjugated secondary antibodies (Thermo Fisher) were used at 1:2000.

VCP inhibitor treatment

DBeQ (Sigma) was dissolved in DMSO to make a 10mM stock solution and diluted 1:1000 in HL3 saline buffer with no calcium (70mM NaCl, 5mM KCl, 10mM $MgCl_2$, 10mM $NaHCO_3$, 115mM sucrose, 4.2mM trehalose and 5mM HEPES) for a final working concentration of 10uM. DMSO was used at the same concentration for the control sample. DBeQ or DMSO were incubated on the dissected muscle for 3 hrs prior to imaging.

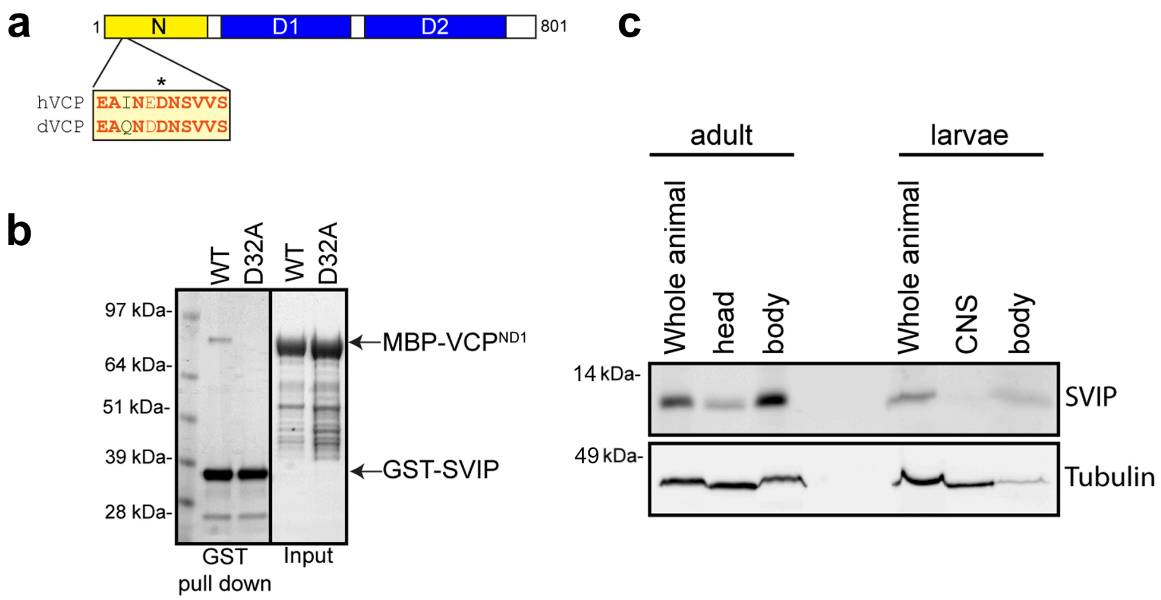

Supplementary Figure 1: SVIP-VCP binding and SVIP expression.

A. Schematic of VCP with relative positions of predicted VIM binding sequence. **B.** In vitro-binding of recombinant MBP-VCP and GST-SVIP fusion proteins. **C.** SVIP protein levels in whole animal, head and body of 3rd instar larvae and adult flies.

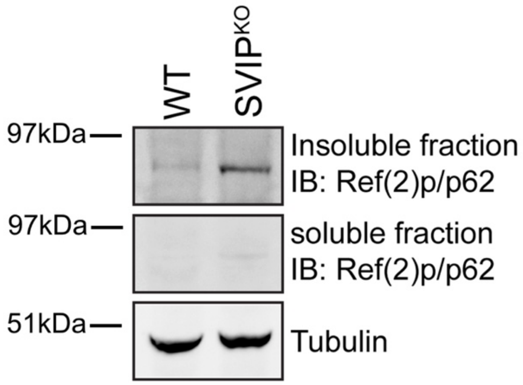

Supplementary Figure 2: SVIP^{KO} have increased insoluble protein fractions.

Insoluble proteins were fractionated by high speed centrifugation (see supplemental methods) and detected by western blot using an ant-Ref(2)p antibody[1]. Tubulin was used as a loading control.

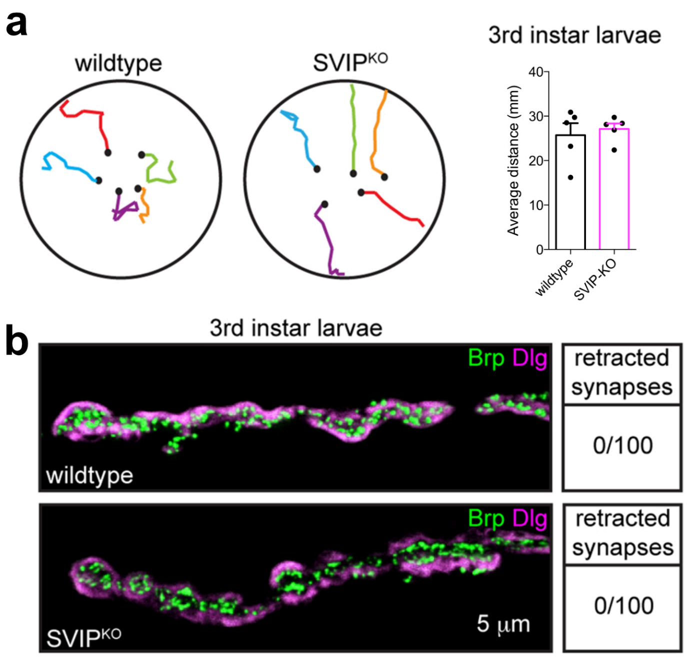

c failure to eclose

genotype	% failure
wildtype	7%
SVIPKO	28%

Supplementary Figure 3: *SVIP^{KO}* phenotypes in 3rd instar larvae.

A. Traces of 3rd instar larvae crawling patterns and quantitation of total distance traveled in 1 min. (n=5 independent animals). Data presented as mean and SEM. **B.** Neuromuscular junctions of 3rd instar larvae stained with the pre-synaptic marker Brp (green) and the post-synaptic marker DLG (magenta). To the right is the quantitated number of retracted synapses observed for 100 synapses. **C.** Failed eclosion rates for *wild type* and *SVIP^{KO}* animals (n=100 for both genotypes).

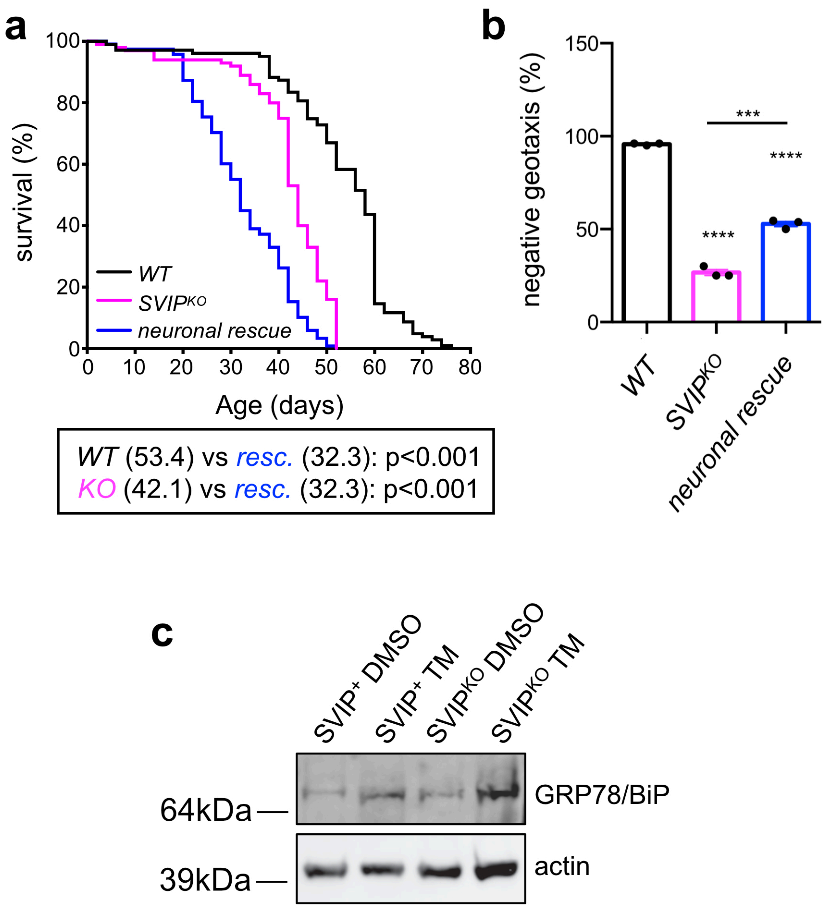

Supplementary Figure 4: Neuronal expression of SVIP fails to rescue SVIP^{KO} mutants.

A. Lifespan analysis of *WT* (n=103), *SVIP^{KO}* (n=100) and neuronal rescue (n=117). **B.** Negative geotaxis assay for the genotypes indicated at 4 weeks of age (n=20 animals scored over 3 independent experiments). Data presented as mean and SEM. Student's T-test for individual comparisons (*** p<0.001, ****p<0.0001). **C.** Western blot analysis of GRP78/Bip protein levels in flies fed tunicamycin for 2 days. Actin was used as a loading control.

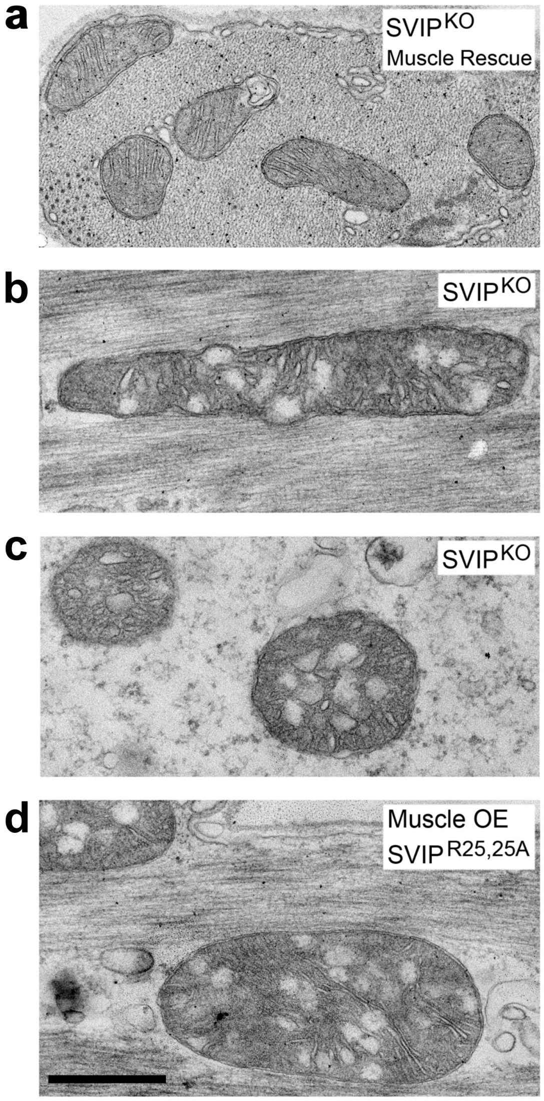

Supplementary Figure 5: Muscle SVIP and SVIP-VCP binding are necessary to sustain mitochondria quality.

A-D. Representative mitochondria in abdominal muscles of 30-day-old animals for the genotypes indicated. Scale bar=1.1μm.

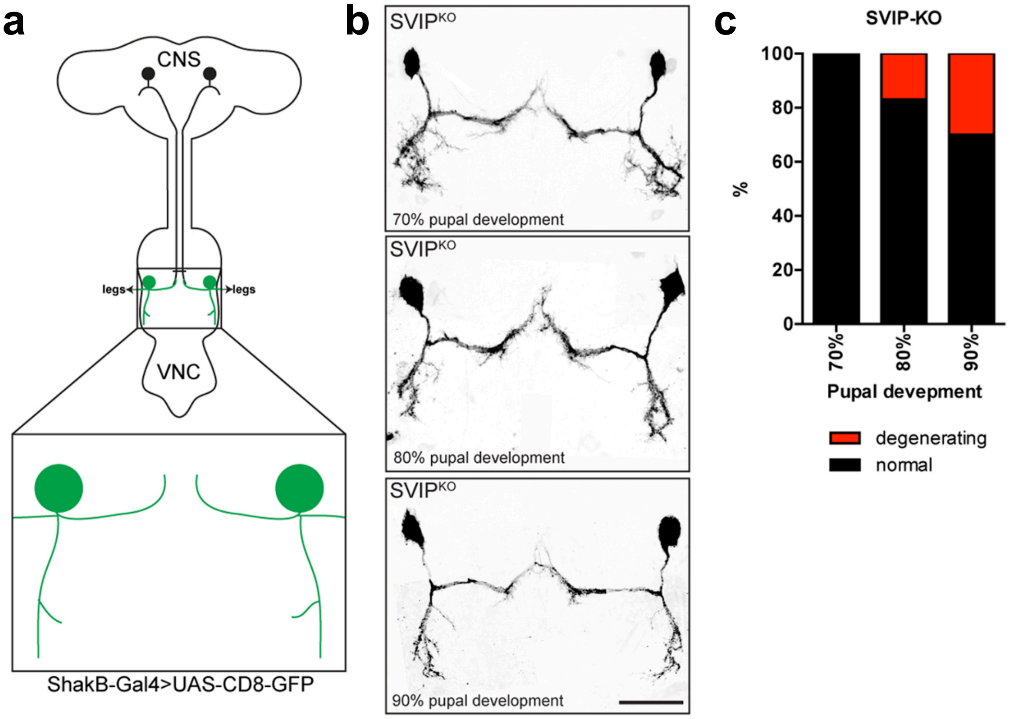

Supplementary Figure 6: TTMn anatomy during pupal development in SVIP^{KO} animals.

A. Schematic of the TTMn anatomy in the ventral nerve cord. TTMn dendrites synapse with GF neurons and project their axons to the leg muscles. **B.** TTMns expressing CD8-GFP (shakB-GAL4, CD8-GFP) were stained with GFP and imaged at 70%, 80% and 90% pupal development. Scale bar=50µm. **C.** Quantitation of degenerating TTMns at each stage of development shown in B (70%, n=10 cells examined over 5 independent animals, 80% n=10 cells examined over 5 independent animals, 90% n=12 cells examined over 6 independent animals).

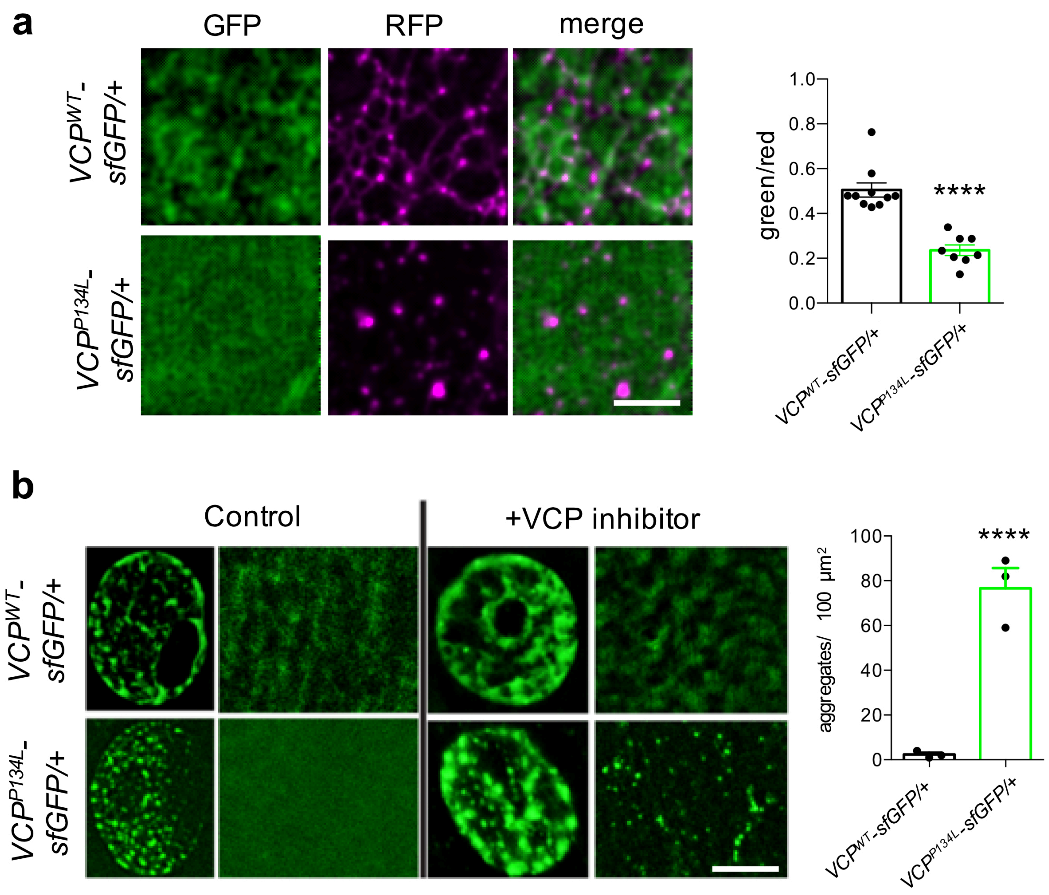

Supplementary Figure 7: VCP^{P134L} is aggregation prone compared to WT VCP.

A. Co-imaging of VCP^{WT}-sfGFP and VCP^{P134L}-sfGFP with Spin-RFP. At right, quantitation of GFP/RFP intensity ratio in RFP masked region. Scale bar=5µm. (*WT*, n=10 muscle cells examined over 5 independent animals. *P134L*, n=8 muscle cells examined over 4 independent animals). **B.** *VCP^{WT}-sfGFP* and *VCP^{P134L}-sfGFP* were expressed endogenously in 3rd instar larval muscles, treated with or without the VCP inhibitor, DBeQ, and the number of fluorescent GFP puncta/100um^2 was quantitated. Scale bar=5µm. (n=3 independent experiments). Data presented as mean and SEM. Student's T-test for individual comparisons (**** p<0.0001).

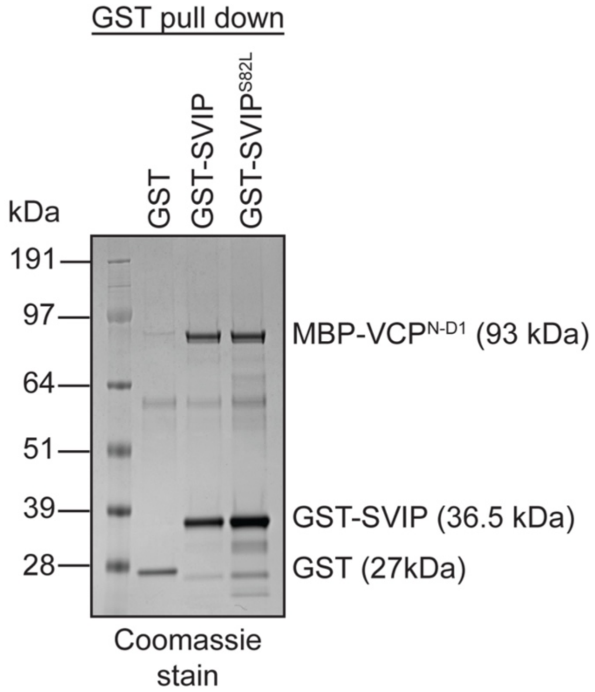

Supplementary Figure 8: SVIP^{S82L} binds to VCP in vitro.

In vitro-binding of recombinant MBP-VCP and GST-SVIP fusion proteins.

Supplementary Table 1

D. melanogaster mutation	Human mutation
P134L	P137L
R152H	R155H
A154R	G157R
R156C	R159C
R188Q	R191Q
L195W	L198W
T259A	T262A
A229E	A232E
N384H	N387H
A436S	A439S

Correspondence of mutations in *Drosophila* and human**.**

Supplementary Table 2 : Primers used in this study

Ter94 CRISPR knock-in

DONOR PLASMID:

Oligos to clone in homology arms:

5' Ter94 5'arm F: GGAGACCTATAGTGTCTTCGGGGCCGA caagtcatcctttgctggagg

5' Ter94 5'arm R: CGTCACAATATGATTATCTTTCTAGGGTTAA AAAGTTCCATTTTGTGGTTTTG

3' Ter94 3'arm F: CGCAGACTATCTTTCTAGGGTTAA ATGAATTTTTTCCGTAAAACCGAGC

3' Ter94 3'arm R: GGAGACGTATATGGTCTTCTTTTCCcgg GCAGAATTGAAGGTGTAGACACTG

Oligos to sequence genomic DNA over target sites:

Ter94 Target1 seqF: CCAACCGACTGATTGTGGAAGAG

Ter94 Target2 seqF: CACCTCAGGATCTGGTAACAAC

Oligos to mutate PAM sequences:

Ter94 PAMmut1 F: GTGTGCATCGTGCTCTCCGACGACACCTGTCCCGAC

Ter94 PAMmut2 F: CCAATCACATTCGCATTCTGGACGCGGTAACTTAC

Oligos to mutate in AarI site before Ter94 stop:

F: GCGACGATGATCTTTACAGTattcGCAGGTGgcagCACCTGCgatcTAGATTTCTAGTTTTACACC

Clone in sfGFP using AarI sites:

sfGFP AarI F: ATTCCACCTGCgcagCAGTGGTGGATCTGGAGGTTCC

sfGFP AarI R: gatcCACCTGCctgcTCTACTTGTACAGCTCATCCATGC

Donor plasmid sequencing oligos:

pHD-SL seq F: CCATCGGTGATGTCGTATAAGAG

pHD-SL-Ter94 seq1: GGGATACGAGGGACTTTATGC

pHD-SL-Ter94 seq2: CCGGCAACCTGTTCGAGATC

pHD-SL-Ter94 seq3: TGGATGGCATGAAGAAGAGC

pHD-SL-Ter94 seq4: CCAACGTGCGCGACATCTTC

pHD-SL-Ter94 seq5: CAGGAAGTACGAGATGTTTGC

pHD-SL-Ter94G seq6: CAACTTCAACAGCCACAACG

pHD-SL-Ter94 seq7: GTCTACGGAGCGACAATTC

pHD-SL-Ter94 seq8: GAAGGGCGAGATCCACAAGG

pHD-SL-Ter94 seq9: CTTACCGCATTGACAAGCAC

pHD-SL-Ter94 seq10: CATTGTCGGTAGAGTGGCTCC

pHD-SL seq R: GAGAGTAGGGAACTGCCAGG

gRNA PLASMID:

gRNA oligos to clone into pCFD4 tandem gRNA plasmid using Gibson:

F oligo (to clone into pCFD4 tandem gRNA plasmid using gibson):
TATATAGGAAAGATATCCGGGTGAACTTCGACGGTGTGCATCGTGCTCTGTTTTAGAGCTAGAAATAGCAAG

R oligo(to clone into pCFD4 tandem gRNA plasmid using gibson):
ATTTTAACTTGCTATTTCTAGCTCTAAAACGGACGCGGTAACTTACTACACGACGTTAAATTGAAAATAGGTC

pCFD4 seq F: GACACAGCGCGTACGTCCTTCG

Supplementary Table 2 (continued)

SVIP CRISPR knock-in

DONOR PLASMID:

Primers to clone in homology arms:

SVIP 5'arm F: ATTCCACCTGCgcagCCGAgtgtgtgcgtaaattgttgagc

SVIP 5'arm R: gatcCACCTGCctgcTTAAATTTTATTTTTGGTTGTACATTTTGGTCG

SVIP 3'arm F: cgtcGCTCTTCtTAAACCAAATTGTTATCAGTGATCGTAAAAATG

SVIP 3'arm R: gacgGCTCTTCcCGGCATTTTCGCTGCTTCTTTCCGTGG

gRNA PLASMID:

Primers to clone guide RNAs into pCFD4 tandem gRNA plasmid using Gibson:

gRNA #1 F: TATATAGGAAAGATATCCGGGTGAACTTCGTTTTGCTGTCACAGCTGAT

GTTTTAGAGCTAGAAATAGCAAG

gRNA #2 R: ATTTTAACTTGCTATTTCTAGCTCTAAAACAATTGTTATCAGTGATCGTCGACGTTAAATTGAAAATAGGTC

SVIP Seq 1 F: CCACGATACGAACAATTAGAACG

Primers to mutate PAM sequences:

SVIP PAMmut#1: cataacagcacgctgttatcGaatcagctgTGACAGC

SVIP PAMmut#2: CTATCTTTCTAGGGTTAAA CgA AATTGTTATCAGTGATCG

Primers to mutate in AarI site before stop:

SVIP AarI mut: ctttcagTGGCAAACTAGCattcGCAGGTGgcagCACCTGCgatcTAAAAGGAAGTGAACATC

Primers to clone in sfGFP using AarI sites:

SVIP sfGFP Gibson F: caacactttgctttcagTGGCAAACTAGCGGTGGATCTGGAGGTTCCGG

SVIP sfGFP Gibson R: CACACCAGGATGGATGTTCACTTCCTTTTACTTGTACAGCTCATCCATGCCCAG

S82L mutagenesis primer:

SVIP S82L KI: gctttcagTGGCAAACTTTATAAAAGGAAGTGAACATC

Supplementary Table 2 (continued)

UAS Expression Vectors

Primers to clone into Donor vectors (gateway):

SVIP attB5 F: GGGG ACA ACT TTG TAT ACA AAA GTT GTA ATGGGAGCCTGTCTGTCCTGCTG

SVIP attB2 R: GGGG AC CAC TTT GTA CAA GAA AGC TGG GTA TTAGCTAGTTTGCCACCTAAGATTGG

SVIP-S82L STOP attB2 R: GGGG AC CAC TTT GTA CAA GAA AGC TGG GTA TTATAAAGTTTGCCACCTAAGATTGGATTG

SVIP attB4r F: GGGG ACA ACT TTT CTA TAC AAA GTT GTA ATGGGAGCCTGTCTGTCCTGCTG

SVIP S82L NS attB3r R: GGGG AC AAC TTT ATT ATA CAA AGT TGT TAAAGTTTGCCACCTAAGATTGGATTG

Primers for 3-fragment gibson into UAS pENTR vector:

FRB-Ter94 F: GGTGGCTCATCTGGCGGAGGTatggcagattccaagggtgaag

FRB-Ter94 R: cttcacccttggaatctgccatACCTCCGCCAGATGAGCCACC

FRB F: CACCatgGAGATGTGGCATGAAGG

Ter94 R: actgtaaagatcatcgtcgccgttg

FKBP-Spin F: CGATGTGGAGCTTCTAAAACTGGAAATGTCGCTGAAACACCAGAAGC

FKBP-Spin R: GCTTCTGGTGTTTCAGCGACATTTCCAGTTTTAGAAGCTCCACATCG

FKBP F: CACCATGGGAGTGCAGGTGGAAACCATCTC

Spin-RA R: ggcaatctgaccgcgctcatcc

Supplementary Table 2 (continued)

Protein Expression Vectors

Primers to clone SVIP into pGEX vector:

SVIP F BamH1: CGCGGATCCATGGGAGCCTGTCTGTCCTGCT

SVIP R Not1: TTTTCCTTTTGCGGCCGCTTAGCTAGTTTGCCACCTAAGATTGGA

SVIP mutagenesis primer:

SVIP R24R25A: ccatcgcctgaggagGCcGCccagcagcagttgga

SVIP S82L NotI R: TTTTCCTTTTGCGGCCGCTTATAAAGTTTGCCACCTAAGATTGGA

Primers to clone VCP into pGEX vector:

Ter94 F BamH1: CGCGGATCCATGGCAGATTCCAAGGGTGAAGATC

Ter94 460R Not1: TTTTCCTTTTGCGGCCGCTTAcgccgatggactggacttggt

VCP mutagenesis primers:

Ter94 R152H F: cgacaactttattgtg**cAc**gccgccatgcgtccta

Ter94 R152H R: taggacgcatggcggcgTgcacaataaagttgtcg

Ter94 R188Q F: gacggtgatccaattaag**cAG**gaggaggaggaggagtcc

Ter94 R188Q R: ggactcctcctcctcctcCTgcttaattggatcaccgtc

Ter94 A291E F: catccatcgctcttcaag**gAG**attggtgtgaagccaccg

Ter94 A291E R: cggtggcttcacaccaatCTccttgaagagcgatggatg

Ter94 L195W F: aggaggaggaggagtccTGGaacgccgttggctacga

Ter94 L195W R: tcgtagccaacggcgttCCAggactcctcctcctcct

Ter94 P134L F: cgagatctatctgaagCTGtacttcctagaggcc

Ter94 P134L R: ggcctctaggaagtaCAGcttcagatagatctcg

Ter94 A154R F: ctttattgtgcgcgcccCGcatgcgtcctattgag

Ter94 A154R R: ctcaataggacgcatgCGggcgcgcacaataaag

Ter94 R156C F: gtgcgcgccgccatgTGTcctattgagttcaag

Ter94 R156C R: cttgaactcaataggACAcatggcggcgcgcac

Ter94 T259A F: ggctgtggccaacgagGccggagcctttttcttcc

Ter94 T259A R: ggaagaaaaaggctccggCctcgttggccacagcc

Ter94 N384H F: gcgcattcacaccaagCacatgaagctgcatgatg

Ter94 N384H R: catcatgcagcttcatgtGcttggtgtgaatgcgc

Ter94 A436S F: gacgataagatcgatTccgaggtgctggcgtcgc

Ter94 A436S R: gcgacgccagcacctcggAatcgatcttatcgtc

Ter94 D32A F: gtggaagaggcgcagaatgacgCcaactctgtggtgtcgc

Ter94 D32A R: gcgacaccacagagttgGcgtcattctgcgcctcttccac

References

1. Pircs, K. *et al.* Advantages and Limitations of Different p62-Based Assays for Estimating Autophagic Activity in Drosophila. *PLoS One* **7**, (2012).
