# Supplementary material for: SVIP is a molecular determinant of lysosomal dynamic stability, neurodegeneration and lifespan
Source: Nat Commun. 2021 Jan 21;12:513. doi: 10.1038/s41467-020-20796-8 (PMC7820495; doi:10.1038/s41467-020-20796-8)
Supplement: Supplementary file 5 — Description of Additional Supplementary Files [file 41467_2020_20796_MOESM5_ESM.docx]

Description of additional supplementary files

Title: Movie S1.

Description: Representative time-lapse imaging of Spin-RFP (lysosomes) in 3rd instar larval Drosophila muscles with SVIPWT over-expression.

Title: Movie S2.

Description: Representative time-lapse imaging of Spin-RFP (lysosomes) in 3rd instar larval Drosophila muscles with SVIPS82L over-expression.
