# Supplementary material for: SVIP is a molecular determinant of lysosomal dynamic stability, neurodegeneration and lifespan
Source: Nat Commun. 2021 Jan 21;12:513. doi: 10.1038/s41467-020-20796-8 (PMC7820495; doi:10.1038/s41467-020-20796-8)
Supplement: Supplementary file 6 — Reporting Summary [file 41467_2020_20796_MOESM6_ESM.pdf]

nature research

Corresponding author(s): Graeme W. Davis

Last updated by author(s): Dec 8, 2020

Reporting Summary

Nature Research wishes to improve the reproducibility of the work that we publish. This form provides structure for consistency and transparency in reporting. For further information on Nature Research policies, see our Editorial Policies and the Editorial Policy Checklist.

Statistics

For all statistical analyses, confirm that the following items are present in the figure legend, table legend, main text, or Methods section.

n/a	Confirmed	
☐	☒	The exact sample size (*n*) for each experimental group/condition, given as a discrete number and unit of measurement
☐	☒	A statement on whether measurements were taken from distinct samples or whether the same sample was measured repeatedly
☐	☒	The statistical test(s) used AND whether they are one- or two-sided<br>*Only common tests should be described solely by name; describe more complex techniques in the Methods section.*
☒	☐	A description of all covariates tested
☐	☒	A description of any assumptions or corrections, such as tests of normality and adjustment for multiple comparisons
☐	☒	A full description of the statistical parameters including central tendency (e.g. means) or other basic estimates (e.g. regression coefficient) AND variation (e.g. standard deviation) or associated estimates of uncertainty (e.g. confidence intervals)
☐	☒	For null hypothesis testing, the test statistic (e.g. *F*, *t*, *r*) with confidence intervals, effect sizes, degrees of freedom and *P* value noted<br>*Give P values as exact values whenever suitable.*
☒	☐	For Bayesian analysis, information on the choice of priors and Markov chain Monte Carlo settings
☒	☐	For hierarchical and complex designs, identification of the appropriate level for tests and full reporting of outcomes
☒	☐	Estimates of effect sizes (e.g. Cohen's *d*, Pearson's *r*), indicating how they were calculated

Our web collection on statistics for biologists contains articles on many of the points above.

Software and code

Policy information about availability of computer code

Data collection	Clampex10.7 (Molecular Devices), Slidebook v5 (Intelligent Imaging Innovations), Micromanager 2.0-gamma (open source)
Data analysis	Prism v.9 (Graphpad), Image J v1.47<br>All statistical analysis were performed using Prism v9 except that the statistical software, OASIS 52, was used to calculate the mean lifespans and perform log-rank tests of lifespans to determine statistical significance.<br>Modeling and analysis of electron micrographs were performed with<br>IMOD v4.9; Images were analyzed in ImageJ 1.47 (http://rsb.info.nih.gov/ij)<br>(http://rsb.info.nih.gov/ij/plugins/time-series.html).

For manuscripts utilizing custom algorithms or software that are central to the research but not yet described in published literature, software must be made available to editors and reviewers. We strongly encourage code deposition in a community repository (e.g. GitHub). See the Nature Research guidelines for submitting code & software for further information.

Data

Policy information about availability of data

All manuscripts must include a data availability statement. This statement should provide the following information, where applicable:

- Accession codes, unique identifiers, or web links for publicly available datasets
- A list of figures that have associated raw data
- A description of any restrictions on data availability

Further information and requests for resources and reagents should be directed to and will be fulfilled by the corresponding author Graeme Davis

(Graeme.Davis@ucsf.edu). All unique/stable reagents generated in this study are available without restriction. This study did not generate datasets for public repositories or new data code.

Field-specific reporting

Please select the one below that is the best fit for your research. If you are not sure, read the appropriate sections before making your selection.

☒ Life sciences ☐ Behavioural & social sciences ☐ Ecological, evolutionary & environmental sciences

For a reference copy of the document with all sections, see nature.com/documents/nr-reporting-summary-flat.pdf

Life sciences study design

All studies must disclose on these points even when the disclosure is negative.

Sample size	A power analysis was performed including estimates of sample size and variation. Sample sizes were also informed by evidence in the published literature and from prior experience where similarly large effect sizes were expected for light microscopy, electrophysiological analysis of synaptic transmission, and electron microscopy. See Johnson et al., 2015 ELife; Genc et al., 2017 ELfe; Hauswirth et al., 2018 ELife. Based on these benchmarks, we estimate that a sample size of 5-10 would provide at least 80% power to detect a difference between control and experimental groups with at least 5% significance level. For lifespan analysis samples sizes exceeded 50 to ensure sufficient statistical power.
Data exclusions	No exclusion criteria were used.
Replication	The numbers of animals and cells for each experiment are stated within the legend of every figure in the paper. Each figure shows the number of data points for each measured parameter.
Randomization	Whenever an experiment compared a treatment or genotype with controls, the experimental and controls samples were prepared in parallel and analyzed in an interleaved manner.
Blinding	Blinding was not possible for experiments in which the genotypes were labeled with visible selection markers, referring to both cellular and organismal assays. Experimenters were not blind to genotype during group allocation because there was no group allocation in our study. Lifespan analyses were not performed blind to genotype because there was no perceived subjectivity to animal death.

Reporting for specific materials, systems and methods

We require information from authors about some types of materials, experimental systems and methods used in many studies. Here, indicate whether each material, system or method listed is relevant to your study. If you are not sure if a list item applies to your research, read the appropriate section before selecting a response.

Materials & experimental systems

n/a	Involved in the study	
☐	☒	Antibodies
☒	☐	Eukaryotic cell lines
☒	☐	Palaeontology and archaeology
☐	☒	Animals and other organisms
☒	☐	Human research participants
☒	☐	Clinical data
☒	☐	Dual use research of concern

Methods

n/a	Involved in the study	
☒	☐	ChIP-seq
☒	☐	Flow cytometry
☒	☐	MRI-based neuroimaging

Antibodies

Antibodies used

Antibodies used:

Primary antibodies:
Ref(2)p, polyclonal, Gabor Juhasz lab (non-commercial)
Tubulin, E7, Developmental Studies Hybridoma Bank, cat#: E7, RRID: AB_2315513
Brp, nc82, Developmental Studies Hybridoma Bank, cat#: nc82, RRID: AB_2314866
SVIP, polyclonal, Cocalico and this study (non-commercial)
VCP, 7F3, Cell Signaling, cat#: 2649
DLG, polyclonal, Vivian Budnik lab (non-commercial)
GFP, 3E6, Thermo Fisher, cat#: 11120
GRP78, 6H4-2G7, Thermo Fisher, cat#: MA5-27687
Actin, ACTN05 (C4), Thermo Fisher, cat#: MA5-11869

Secondary antibodies:

	Alexa Fluor® 488 AffiniPure Goat Anti-Mouse IgG (H+L), Jackson Immuno-research Laboratories, Cat #:115-545-003, RRID: AB_2338840<br>Cy™3 AffiniPure Goat Anti-Rabbit IgG (H+L), Jackson Immuno-research Laboratories, Cat #:111-165-003, RRID: AB_2338000<br>Goat anti-Rabbit IgG (H+L) Secondary Antibody, HRP, Thermo Fisher, Cat#: 65-6120, RRID: AB_2533967<br>Goat anti-Mouse IgG (H+L) Secondary Antibody, HRP, Thermo Fisher, Cat#: 31430, RRID: AB_228307
Validation	Validation for commercial antibodies can be found on the following websites:<br>Tubulin: https://dshb.biology.uiowa.edu/E7_2<br>Brp: https://dshb.biology.uiowa.edu/nc82<br>VCP: https://www.cellsignal.com/products/primary-antibodies/vcp-7f3-rabbit-mab/2649<br>GFP: https://www.thermofisher.com/antibody/product/GFP-Antibody-clone-3E6-Monoclonal/A-11120<br>GRP78: https://www.thermofisher.com/antibody/product/GRP78-Antibody-Polyclonal/PA5-22967<br>Actin: https://www.thermofisher.com/antibody/product/Actin-Antibody-clone-ACTN05-C4-Monoclonal/MA5-11869<br>Alexa Fluor® 488 Goat Anti-Mouse: https://www.jacksonimmuno.com/catalog/products/115-545-003<br>Cy™3 Goat Anti-Rabbit: https://www.jacksonimmuno.com/catalog/products/111-165-003<br>Goat anti-rabbit, HRP: thermofisher.com/antibody/product/Goat-anti-Rabbit-IgG-H-L-Secondary-Antibody-Polyclonal/65-6120<br>Goat anti-mouse, HRP: https://www.thermofisher.com/antibody/product/Goat-anti-Mouse-IgG-H-L-Secondary-Antibody-Polyclonal/31430

Animals and other organisms

Policy information about studies involving animals; ARRIVE guidelines recommended for reporting animal research

Laboratory animals	all experiments were performed on male and female Drosophila melanogaster. Third instar larvae were used. All adult animal ages are specifically defined (lifespan and degeneration). Age of pupal development is specified for each stage analyzed.
Wild animals	no wild animals were used in this study
Field-collected samples	no field collected animals or samples were used in this study
Ethics oversight	No ethical oversight was necessary for studies of invertebrate Drosophila animals.

Note that full information on the approval of the study protocol must also be provided in the manuscript.
